# Negative symptomatology and clozapine-induced obsessive–compulsive symptoms: a cross-sectional analysis

**DOI:** 10.1007/s00406-025-02021-z

**Published:** 2025-05-16

**Authors:** Phillip Kleymann, Carla Morgenroth, Stefan Gutwinski, Felix Bermpohl, Daniel Schulze, Elias Wagner, Alkomiet Hasan, Cynthia Okhuijsen-Pfeifer, Jurjen J. Luykx, Marte Z. van der Horst, Tatiana Oviedo-Salcedo, Stefanie Schreiter

**Affiliations:** 1 Department of Psychiatry and Neurosciences, Charité Universitätsmedizin Berlin, Corporate Member of Freie Universität Berlin and Humboldt-Universität Zu Berlin, Berlin, Germany; 2 Department of Psychiatry and Psychotherapy, St. Hedwig-Krankenhaus, Charité-Universitätsmedizin, Berlin, Germany; 3 Institute of Biometry and Clinical Epidemiology, Charité Universitätsmedizin Berlin, Corporate Member of Freie Universität Berlin and Humboldt-Universität Zu Berlin, Berlin, Germany; 4 Department of Psychiatry, Psychotherapy and Psychosomatics, University of Augsburg, Augsburg, Germany; 5 Evidence-Based Psychiatry and Psychotherapy, Faculty of Medicine, University of Augsburg, Augsburg, Germany; 6 DZPG (German Center for Mental Health), Partner Site München/Augsburg, Augsburg, Germany; 7 Department of Psychiatry, University Medical Center Utrecht, Utrecht University, Utrecht, The Netherlands; 8 Department of Psychiatry and Psychotherapy, University Hospital-LMU Munich, Munich, Germany

**Keywords:** Schizophrenia, Clozapine, Obsessive–compulsive symptoms, Negative symptomatology, Moderated regression analysis

## Abstract

Obsessive–compulsive symptoms (OCS) frequently manifest in individuals with schizophrenia, affecting their prognosis and quality of life. The etiology of OCS in schizophrenia is complex, with theories ranging from subtype-specific manifestations to pharmacological influences. Notably, clozapine has been associated with a higher prevalence of OCS. However, the clinical factors influencing clozapine-induced OCS remain unclear. This cross-sectional study recruited individuals diagnosed with schizophrenia who were using clozapine, as well as a comparison group of individuals diagnosed with schizophrenia who were using other second-generation antipsychotics (SGA). Clinical assessments included OCS which were quantified using the Obsessive–Compulsive Inventory-Revised (OCI-R). 189 Participants were recruited, of whom 129 were taking clozapine and 60 other atypical antipsychotics. Statistical analyses, including moderated regression modeling, identified clinical factors influencing OCS occurrence. Clozapine users exhibited significantly higher OCI-R scores compared to non-clozapine users (p = 0.001). Moderated regression analysis revealed a moderating effect of negative symptom severity, indicating that when negative symptoms increased, the difference in OCI-R scores between clozapine and non-clozapine groups decreased. Other factors like duration of illness, medication duration, and psychopathology severity did not significantly moderate the group differences in OCI-R scores. As negative symptoms worsened, the impact of clozapine on OCS lessened, a pattern not seen with other antipsychotics. This suggests that clozapine’s effect on OCS is specific and influenced by different mechanisms. The study recommends screening for OCS in patients with mild negative symptoms and further research into biological markers to better understand clozapine-induced OCS.

**Supplementary Information:**

The online version contains supplementary material available at 10.1007/s00406-025-02021-z.

## Introduction

People with schizophrenia (PwS) frequently show obsessive–compulsive symptoms (OCS) or obsessive–compulsive disorders (OCD). According to a meta-analysis, the mean OCS prevalence in PwS was 30.7% and 12.3% for OCD [1]. These symptoms are often accompanied by a worsened prognosis, higher suicidality rate [2–4], more severe depressive symptoms [2, 3] and a poorer quality of life [5].

Different theories regarding the etiology of OCS in PwS are under discussion: some propose a certain OCD-subtype of schizophrenia. Notably, a significant proportion of PwS experience OCS (30.7%), contrasting with the relatively low occurrence (1.7%) of psychotic symptoms among all patients diagnosed with OCD [6]. This has prompted discussions around the concept of a “schizo-obsessive” subtype of psychosis [7–10].

Other evidence points in the direction of pharmacologically induced OCS. The potential of clozapine to induce OCS was first observed by Den Haag et al. [11] and Baker [12] in 1992. Since then, several studies and reviews have suggested that second-generation antipsychotics (SGA) can induce OCS in PwS, especially clozapine. According to two different systemic reviews, risperidone demonstrates a de-novo OCS rate of approximately 3%, while olanzapine exhibits a higher de novo OCS rate ranging between 11 and 20%, and clozapine surpasses both with a de novo OCS rate falling within the range of 20–28% [13, 14].

Despite the occurrence of different serious side effects, clozapine is still a valuable treatment option. Meta-analyses and registry studies have demonstrated that the effectiveness of clozapine in treatment resistant patients surpasses that of other SGA [5, 15, 16]. Consequently, clozapine is considered the pharmacological treatment of choice for individuals with treatment-resistant schizophrenia [17], but is still underprescribed [18].

The mechanisms underlying OCS in PwS and especially in patients taking clozapine is not understood. Therefore, it is crucial to discern the factors that increase the risk of clozapine-induced OCS as well as those that mitigate it. The development of OCS under clozapine therapy may also be influenced by genetic factors. In a review, Schirmbeck et al. proposed that clozapine-induced OCS might be conceptualized as resulting from gene-environment interactions [19]. Different potential risk genes have been discussed in the literature, although future research is warranted [19]. However, a genome-wide association study by Morgenroth et al. did not identify a specific risk gene; rather, it found a potential correlation between OCD phenotype and the polygenic risk score for clozapine metabolism [20].

Several risk factors for clozapine-induced OCS are discussed in the literature like duration of illness and duration of treatment, clozapine blood levels or symptom severity. The duration of illness had no influence on the severity of OCS according to a naturalistic cross-sectional survey by Schreiter et al. [21], but a correlation with higher OCS could be demonstrated in several studies for the duration of clozapine medication [21–23], with the exception of one study [24]. Many studies have shown that higher blood doses of clozapine are correlated with clozapine-induced OCS [12, 23–25].

In various studies, a correlation between the severity of symptoms of schizophrenia and the manifestation of OCS has been observed during clozapine treatment [22, 26, 27]. Schirmbeck [23] showed in a cross-sectional study that clozapine and olanzapine users had higher rates of OCS than the comparison group of aripiprazole and amisulpride users. These higher scores did correlate with lower cognitive test scores [23]. Some studies have shown a correlation between depressive symptoms and elevated OCS in patients on clozapine treatment [26–28]. Only one retrospective cross-sectional study could not find this association [24].

To our knowledge, smoking as a potential clinical co-factor has only been investigated in one cross-sectional study with patients undergoing a clozapine therapy: Bria [24] found no correlation between current smoking and OCS.

In summary, there is little knowledge on clinical factors that may have an influence on the occurrence or the worsening of OCS under a clozapine therapy. Further, various studies lack a comparison group of non-clozapine users. The control group of this study, which did not undergo clozapine treatment, allows the conclusion to be drawn that potential moderating effects on clozapine-induced OCS are specific to individuals treated with clozapine. Consequently, potential moderating influences on OCS indicate an association with clozapine use, although OCS occur comorbidly in schizophrenia independently of clozapine treatment. The primary goal of this study was to shed light on possible clinical factors and their impact on clozapine-induced OCS in a cohort of clozapine and other SGA treated patients. The potential clinical impact factors, examined in this study were duration and severity of illness, clozapine dosage and duration of clozapine medication, depressive symptoms, global functioning, smoking behavior, and cognitive function. By gaining insight into the potential risk factors associated with this adverse effect of clozapine, it would be possible to enhance our comprehension of the matter and to identify individuals at high risk for further screening.

## Methods

### Study sample

This study was part of a larger, international multicenter cohort study (Clozin Consortium) led by the University Medical Center Utrecht. The consortium’s primary objectives are to investigate the underlying genetic architecture of treatment-resistant Schizophrenia, identify clinical and genetic predictors of clozapine effectiveness and examine the occurrence of side effects [29].

The sub-sample for the current study was recruited at the Department of Psychiatry and Neurosciences at Charité Berlin, the Department of Psychiatry and Psychotherapy at University Hospital of Munich (LMU Munich), and through advertisement throughout the city of Berlin between May 2017 and May 2023. The study population was divided into two groups: one group was currently on clozapine treatment, while the control group was receiving a different SGA. Inclusion criteria defined for this population were (1) a diagnosis of schizophrenia, schizophreniform disorder, schizoaffective disorder, or psychosis not otherwise specified (NOS) (2) current medication with an SGA for at least 6 weeks, (3) age of at least 18 years, (4) sufficient German language skills, and (5) mental competency to decide about his/her participation in this study. Exclusion criteria were: (1) Lack of capacity to consent or hospitalization against the patient’s will and (2) a history of Parkinson’s disease. All patients gave written and informed consent prior to participating in this study. The study was approved by Charité’s ethic committee (Reference number: EA1/056/20).

### Clinical assessment and instruments

A standardized interview was conducted with each participant, gathering sociodemographic and clinical information, such as history of illness, medication and substance use. To compare dosages among different SGAs, the olanzapine equivalent based on defined daily dosages by Leucht et al. was utilized [30].

The study’s endpoints were the severity of OCS and the occurrence of OCD. OCS and OCD were quantified using the revised version of the Obsessive–Compulsive Inventory—Revised (OCI-R). The OCI-R is a self-report scale, measuring the major symptoms in six different groups of OCS [31].

To quantify the covariates, a battery of psychometric tests was performed on the study population to test different psychiatric symptoms. To measure overall well-being, the Clinical Global Impression scale (CGI-S) [32] and the Global Assessment of Functioning (GAF) [33] were performed. The Positive and Negative Syndrome Scale (PANSS) and its negative positive and general subscale were used to quantify the severity of symptoms of schizophrenia [34]. The Calgary Depression Scale for Schizophrenia (CDSS) assesses the level of depression [35]. The Fagerström Test for Nicotine Dependence (FTND) was used as a standard instrument for evaluating the intensity of physical nicotine addiction [36]. To assess cognitive flexibility, the Trail-Making-Test was utilized in this study [37, 38]. Where possible, missing data were supplemented from the hospital information system.

### Statistical analyses

We conducted statistical analyses using IBM SPSS Statistics v.27 with the Process Add-on v.4.2 by Andrew Hayes [39]. A comparison of demographic, psychological test scores, and medical history was conducted between the two groups using either independent t-tests, Chi^2^-test, or Mann–Whitney-U tests, as appropriate.

**Fig. 1:**
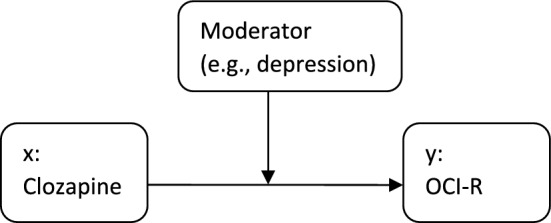
Statistical model for the primary research question

To identify potential clinical impact-factors on the occurrence of OCS, we used a moderated regression-model, performed with the Process Add-On v.4.2 in SPSS by Andrew Hayes [39]. Moderated regression-model was employed to analyze if clinical factors had a moderating effect on the differences in the OCI-R scores between the two groups. Each clinical factor was analyzed separately, with the significance level set at 0.05 (see Fig. 1).

In order to prevent potential influence of confounders, covariates were included in the regression analysis that had a potential influence on either the level of the OCI-R or on the classification into one of the two groups (Clozapine vs. Non-Clozapine). Included covariates were (1) whether a participant had taken antidepressants or not (dichotomous variable), because antidepressants have been shown to alleviate OCS [40] and (2) the duration and severity of illness (continuous variable), as it is more likely that long and/or more severe illness is found in clozapine users, because clozapine is only used in treatment resistant schizophrenia [17]. If a significant difference was found for a clinical factor in the group comparison, it was also included as a covariate in the moderated regression analysis.

## Results

### Clinical characteristics of the sample

One hundred and seventy-nine participants were recruited. 129 of them underwent clozapine therapy and 60 of them were taking other SGAs. Of the participants in the clozapine group, 63 (48.83%) were taking an additional SGA as comedication (21 were taking aripiprazole, 25 amisulpride, 5 olanzapine, 4 paliperidone, 16 risperidone and 4 quetiapine). Therefore, 66 (51.17%) participants had just received clozapine therapy. Of the participants undergoing SGA treatment, 16 were taking aripiprazole, 19 amisulpride, 3 cariprazine, 16 olanzapine, 5 paliperidone, 9 risperidone and 6 quetiapine. The sum of these numbers exceeds 60, as 12 (20%) participants in the SGA group were undergoing therapy with more than one SGA, 48 (80%) participants were taking just one SGA. Differences in sociodemographic and clinical characteristics of the two groups are described in Table 1. Patients on clozapine therapy had significantly higher OCI-R scores than those receiving another SGA therapy (p = 0.001). The group that was treated with clozapine had a significantly longer duration of illness (p = 0.001) and duration of current medication (p = 0.014). The clozapine group also had significantly higher disease severity, as measured by the CGI-S (p = 0.022), and significantly lower level of functioning, as measured by the GAF (p = 0.025). In contrast, the PANSS scores indicated no notable discrepancies in symptom levels. The group treated with clozapine took significantly longer to complete the TMT-B (p = 0.001), which may indicate attention deficit and executive dysfunction. This significant difference is not present in TMT-A. The two groups differed significantly in terms of the schizophrenic subtype (p = 0.005), as the SGA group was composed exclusively of individuals diagnosed with schizophrenia. In contrast, the Clozapine group included individuals diagnosed with schizophrenia (84%) as well as schizophrenic disorder or schizoaffective disorder (together 85.3%). We also controlled for other comedication. No significant differences were observed between the clozapine and non-clozapine groups in the number of patients taking antidepressants, mood stabilizers, or benzodiazepines.

**Table 1:** Description of clinical factors between the clozapine and non-clozapine group

	Clozapine Mean (SD)/n (frequency) n = 129	Non-clozapine Mean (SD)/n (frequency) N = 60	Test statistics
Female participants	47 (36.4%)	19 (31.7%)	p = 0.522 Χ^2^ = 0.410
Age in years	44.02 (± 11.76)	41.85 (± 11.34)	p = 0.200 Z = ** − **1.280
No. of participants with Schizophrenia (ICD-10 F20.x)	109 (84.5%)	60 (100%)	p = 0.001* **Χ**^**2**^** = 10.043**
No. of participants with schizoaffective disorder (ICD-10 F25.x)	19 (14.7%)	0 (0%)	p = 0.001* **Χ**^**2**^** = 10.043**
No. of participants with schizophrenic disorder (ICD-10 F20.8)	1 (0.8%)	0 (0%)	p = 0.001* **Χ**^**2**^** = 10.043**
Duration of illness in months	18.6 (± 11.6)	12.1 (± 7.9)	p = 0.001* **Z = − 3.435**
Duration of main SGA^a^ in months	10.28 (± 9.33)	6.34 (± 5.88)	p = 0.014* **Z = − 2.457**
Olanzapine equivalent (in mg)	7.8 (± 5.3)	8.8 (± 5.0)	p = 0.294 Z = ** − **1.050
No. of participants taking 1 SGA	66 (51.16%)	48 (80.00%)	p = 0.001* **t = 4.668** **CD = 0.610**
No. of participants taking 2 SGA	54 (41.86%)	12 (20.00%)	p = 0.002* **t = − 3.219** **CD = − 0.487**
No. of participants taking 3 or more SGA	9 (7.00%)	0 (0.00%)	p = 0.002* **t = − 3.098** **CD = − 0.310**
No. of SGA taken	1.56 (± 0.40)	1.20 (± 0.64)	p = 0,001* **t = − 4,683** **CD = − 0.625**
No. of participants taking antidepressants	32 (24.03%)	11 (16.67%)	p = 0.324 Z = ** − **0.985
No. of participants taking mood stabilizers	19 (27.13%)	15 (25%)	p = 0.220 Z = ** − **1.226
No. of participants taking benzodiazepines	20 (14.72%)	5 (8.33%)	p = 0.177 Z = ** − **1.351
OCI-R mean score	14.58 (± 10.70)	8.85 (± 8.85)	p = 0.001* **t = − 3.427** **CD = − 0.595**
PANSS total	63.60 (± 16.46)	61.35 (± 17.01)	p = 0.402 Z = ** − **0.837
PANSS positive	15.37 (± 5.32)	15.78 (± 6.45)	p = 0.892 Z = ** − **0.136
PANSS negative	15.86 (± 5.57)	15.65 (± 5.54)	p = 0.808 t = ** − **0.243
PANSS general	32.43 (± 8.58)	30.43 (± 8.10)	p = 0.206 Z = ** − **1.265
CDSS	4.32 (± 4.11)	5.52 (± 5.05)	p = 0.087 t = 1.719
FTDN	3.02 (± 3.31)	3.31 (± 3.40)	p = 0.588 t = 0.543
GAF	51.78 (± 13.89)	59.7 (± 18.56)	p = 0.004* **t = 2.930** **CD = 0.512**
CGI-S	4.24 (± 1.04)	3.93 (± 1.22)	p = 0.009* **t = − 0.454** **CD = − 0.413**
TMT-A in seconds	58.00 (± 81.55)	43.5 (± 29.48)	p = 0.119 t = ** − **1,289
TMT-B in seconds	133.09 (± 86.72)	95.51 (± 43.33)	p = 0.001* **t = − 3.497** **CD = − 0.501**

*CD* Cohen’s d, *CGI-S* Clinical Global Impression Scale, *CDSS* Calgary Depression Scale for Schizophrenia, *FTDN* Fagerström Test for Nicotine Dependence, *GAF* Global Assessment of Functioning, *OCI-R* Obsessive–Compulsive Inventory—Revised, *PANSS* Positive and Negative Syndrome scale, *SGA* Second Generation Antipsychotic, *TMT* Trail-making test

Test*-*Statistics of group comparison are presented, testing with t-tests (if normally distributed) or Mann–Whitney-U-tests (if not normally distributed) or Chi^2^-Test (if variables were categorial)

^a^Clozapine. If not taken, the SGA with the highest Olanzapine equivalent

* and bold *p* < 0.05

### Results of moderated regression analysis

Severity of illness measured by the CGI-S, duration of illness, whether a participant is treated with antidepressants or not, and the TMT-B were included as covariates due to their significant group difference and/or influence on the OCI-R. The GAF, as well as the duration of current medication were *not* included as covariates, despite a group difference being found, as these variables are each too similar to the already included covariates.

**Fig. 2:**
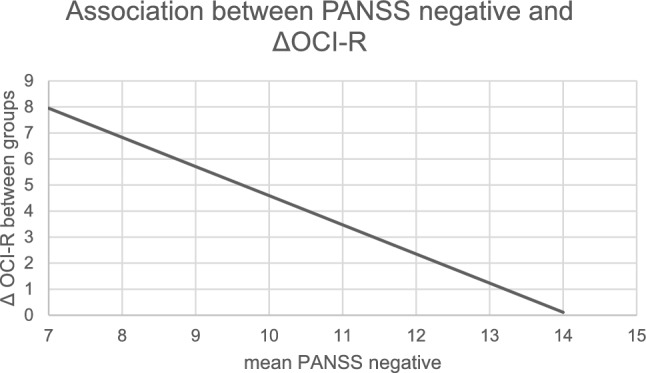
Association between PANSS-negative and the group difference in OCI-R. The y-axis describes the difference between the clozapine and the non-clozapine group in the OCI-R Score calculated in the moderated regression analysis. The x axis describes the PANSS-negative score. Seven is the minimum score in the PANSS negative scale, that is why the x-axis has no lower values. *OCI-R* Obsessive–compulsive inventory—revised, *PANSS* Positive and Negative Syndrome Scale

The regression model revealed a moderating effect of the negative PANSS score as a clinical factor (− 1.12 [− 1.76 to − 0.49]). The difference between the Clozapine vs non-clozapine-group in the OCI-R level decreases with higher negative symptoms, as measured with the PANSS negative score. Put differently, with increasing PANSS negative scores, the difference between the groups on OCI-R scores became smaller until it disappeared completely. Table 2 presents the findings of the moderated regression analysis. The displayed coefficients illustrate the theoretical increase or decrease in the regression model on the OCI-R score when a point is added on the respective clinical cofactor. The moderation is illustrated in Fig. 2, which depicts the group difference between the two groups with respect to the OCI-R in relation to the PANSS negative. As the PANSS negative decreases, the group difference increases. More in-depth results of moderated regression analysis with PANSS-negative subscale can be found in the Online Resource 1.

**Table 2:** Results of the moderated regression analysis with and without covariates for all clinical factors studied

Clinical co-factor/coefficient	Regression coefficient without covariates [95%-CI]	Regression coefficient with covariates ^a^ [95%-CI]
Duration of illness (in months)	0.09 [− 0.28 to 0.46]	0.14 [− 0.25 to 0.53]
Duration of main SGA^b^ medication (in months)	0.18 [− 0.85 to 1.20]	− 0.05 [− 0.97 to 0.87]
Olanzapine equivalent (in mg)	0.125 [− 0.57 to 0.82]	0.10 [− 0.67 to 0.86]
Antidepressant medication	0.37 [− 8.16 to 8.91]	− 1.51 [− 9.90 to 6.88]
Mood stabilizers	− 8.64 [− 19.34 to 2.07]	−0.54 [− 13.76 to 12.68]
Benzodiazepines	− 2.13 [− 13.35 to 9.09]	0.04 [12.91 to 12.99]
CGI	− 0.95 [− 3.68 to 1.79]	− 2.37 [− 5.63 to 0.88]
GAF	− 0.05 [− 0.24 to 0.15]	0.03 [− 0.21 to 0.26]
PANSS total	− 0.11 [− 0.29 to 0.09]	− 0.20 [− 0.43 to 0.03]
PANSS positive	0.09 [− 0.43 to 0.62]	− 0.17 [− 0.86 to − 0.52]
PANSS negative	− **0.83 [**− **1.40** to − 0.27]*	− **1.12 [**− **1.76 to** − 0.49]*
PANSS general	− 0.14 [− 0.51 to 0.23]	− 0.25 [− 0.69 to 0.20]
CDSS	− 0.63 [− 1.34 to 0.08]	− 0.51 [− 1.31 to 0.28]
TMT A	0.08 [− 0.03 to 0.19]	0.05 [− 0.06 to 1.70]
TMT B	− 0.05 [− 0.12 to 0.01]	− 0.05 [− 0.12 to 0.01]
FTDN	− 0.05 [− 0.12 to 0.01]	0.05 [− 1.02 to 1.11]

Regression coefficient: the difference in OCI-R, that is added when one point of the clinical factor is added, shown with and without covariates being considered. To illustrate, an increase of one point in the calculated model for a cofactor will result in a corresponding increase in the OCI-R score by the stated value

*CDSS* Calgary Depression Scale for Schizophrenia, *CI* Confidence interval, *CGI-S* Clinical Global Impression scale, *FTDN* Fagerström Test for Nicotine Dependence, *GAF* Global Assessment of Functioning, *OCI-R* Obsessive–Compulsive Inventory—Revised, *PANSS* Positive and Negative Syndrome scale, *TMT* Trail-making test

^a^Covariates: Duration, CGI, TMT-B, and Antidepressant

^**b**^**Clozapine. If not taken, the SGA with the highest olanzapine equivalent**

^*^and bold p < 0.05

## Discussion

### Main finding

Our data showed a significant association between clozapine treatment and more severe OCS, consistent with previous research [13, 14]. These findings underscored the importance of carefully monitoring OCS in patients with schizophrenia who are treated with clozapine.

More importantly, our study revealed that this difference in OCS between clozapine users and non-clozapine users diminishes as the severity of negative symptoms increases. The impact of clozapine on OCS is more pronounced when negative symptoms are less severe. For instance, individuals with low PANSS negative scores who are taking clozapine tend to have higher OCI-R scores compared to those not on clozapine. However, among individuals with high PANSS negative scores, this difference in OCI-R scores becomes negligible. This effect was observed exclusively in the context of PANSS negative symptoms. The scores of PANSS positive did not exert a moderating influence on the OCI-R scores.

This pattern suggests that the relationship between clozapine use, and OCS may be moderated by the severity of negative symptoms: clozapine’s association with higher OCS scores is most evident in patients with milder negative symptoms. Notably, this moderating effect appears to be only seen in the clozapine group, as no similar pattern was observed in the control group treated with other atypical antipsychotics. This indicates that the observed relationship between negative symptoms and OCS is unique to those receiving clozapine, rather than a general feature of schizophrenia.

Three potential explanations may account for our findings: First, clozapine's effects on positive symptoms, negative symptoms, and the development of OCS are likely mediated by different mechanisms. The reduction of positive symptoms is thought to involve antagonism of dopamine D2 and D4 receptors, [41, 42], while its effect on negative symptoms is likely associated with serotonin 5-HT2 A receptors blockade [42–44].

Among SGAs, clozapine's high affinity for serotonin receptors is hypothesized to underlie its strong association with OCS, suggesting a primarily serotonergic mechanism. However, dopaminergic and glutamatergic pathways may also contribute, though evidence remains limited [45]. The interplay between these pathways and clozapine’s pharmacological profile likely explains its nuanced effects on positive symptoms, negative symptoms, and OCS.

Given this, it is possible that clozapine’s impact on negative symptoms and OCS may share overlapping serotonergic pathways, whereas its effects on positive symptoms are mediated by distinct dopaminergic mechanisms. This could explain the observed moderating role of negative symptoms in the relationship between clozapine use and OCS severity. Patients with a stronger response of negative symptoms to clozapine may be at higher risk of developing OCS, though further research is needed to confirm this hypothesis.Another explanation is that individuals with severe negative symptoms may have a reduced tendency to develop clozapine-induced OCS. Altered prefrontal-striatal circuits, which are implicated in negative symptoms [46], might interact with the mechanisms underlying OCS, resulting in a lower susceptibility to clozapine’s serotonergic effects in this subgroup. However, this hypothesis is speculative and warrants further exploration.

A third explanation may be that elevated negative symptoms might obscure the presence of OCS due to cognitive impairments and diminished self-awareness in affected individuals. This underreporting could contribute to an underestimation of OCS in patients with pronounced negative symptoms.

### Limitations

Limitations of this study include the composition of the comparison group, which solely comprised of patients diagnosed with schizophrenia, while the clozapine group also comprised patients with schizoaffective or schizophreniform disorders, (14.3% and 1.1% respectively). The cross-sectional nature of the study also introduces constraints, notably the inability to track the development of OCS over time. Prior research indicates a correlation between the duration of clozapine treatment and the intensity of OCS [21–23], raising the question of causality versus exacerbation of pre-existing conditions. The absence of baseline data on OCS/OCD, positive and negative symptoms prior to the initiation of clozapine or SGA therapy limits our ability to definitively attribute these symptoms to the medication. Future research should employ a longitudinal approach to circumvent these limitations. A further limitation of the study is the heterogeneity in antipsychotic medication in the two groups. A more pharmacologically stringent approach could be to include only patients who undergo antipsychotic monotherapy or to take into account the fact that SGAs other than clozapine, such as olanzapine or risperidone, also trigger OCS [13]. The inclusion of patients receiving antidepressants, mood stabilizers, and benzodiazepines reflects the high prevalence of comorbidities and polypharmacy in real-world schizophrenia populations. Importantly, there were no significant differences between the clozapine and non-clozapine groups in the use of these adjunctive medications, supporting the comparability of the groups. However, the serotonergic effects of antidepressants could have biased the results toward more conservative estimates, potentially masking clozapine-induced OCS. The olanzapine equivalent utilized in this study does reflect the serotonergic and dopaminergic effects. However, it still lacks full representation of the glutamatergic effects, which may play a vital role in the development of OCS. Additionally, future studies could benefit from a foreign-rated scale for the assessment of OCS, such as the Yale-Brown Obsessive–Compulsive Scale, the assessment of clozapine serum levels, and a more detailed analysis of negative symptoms (e.g., using the Mader method).

## Conclusion

In conclusion, our data suggests that the severity of negative symptoms in PwS may be associated with the occurrence of OCS. Individuals exhibiting mild negative symptoms may be at an elevated risk for the development of clozapine-induced OCS. Although the precise mechanisms responsible for this potential relationship remain elusive, it may be beneficial to introduce screening for negative symptoms as well as OCS when clozapine is used. In PwS with particularly mild negative symptoms, it might be advisable to be particularly vigilant regarding the development of OCS. However, more research is needed to identify and investigate risk factors to better determine which patients are at an increased risk of developing OCS while taking clozapine. To improve the prevention of clozapine-induced OCS, further markers, in particular biological markers, would be desirable. The study by Morgenroth et al., for example, attempted to identify risk genes that may favor OCS under clozapine therapy [20]. There is evidence to suggest that other SGAs, such as olanzapine or risperidone, may also induce OCS [13]. Further investigation into this phenomenon is indicated.

## Supplementary Information

Below is the link to the electronic supplementary material.

Supplementary file1 (DOCX 24 KB)

## Data Availability

Raw data for all datasets are not publicly available to preserve individuals’ privacy under the European General Data Protection Regulation.
