# Supplementary material for: Negative symptomatology and clozapine-induced obsessive–compulsive symptoms: a cross-sectional analysis
Source: Eur Arch Psychiatry Clin Neurosci. 2025 May 16;276(3):991–9. doi: 10.1007/s00406-025-02021-z (PMC13002749; doi:10.1007/s00406-025-02021-z)
Supplement: Supplementary file 1 — Supplementary file1 (DOCX 24 KB) [file 406_2025_2021_MOESM1_ESM.docx]

Table S1: Moderated regression analysis results of the moderated regression analysis with PANSS-negative subscale

|  | Without covariates | |  | With covariates^a^ | |
| --- | --- | --- | --- | --- | --- |
|  | **Coeff [95%-CI]** | ***p*** |  | **Coeff [95%-CI]** | ***p*** |
| **constant** | 3.27  [-1.99 - 8,52] | 0.2213 |  | 2.25  [-9.15 – 13.65] | 0.6959 |
| **Group difference** | 5.74  [2,51 - 8,98] | 0.0006* |  | 3.85  [0.09 – 7.62] | 0.0451* |
| **PANSS negative** | 1.47  [0.52 - 2,41] | 0.0026* |  | 1.59  [0.55 – 2.64] | 0.0451* |
| **Group * PANSS negative** | -0.83  [-1.40 – -0.27] | 0.0043* |  | -1.12  [-1.76 – -0.49] | 0.0007* |
| **Duration of illness** |  |  |  | -0.01  [-0.18- 0.16] | 0.8964 |
| **CGI-S** |  |  |  | 1.96  [0.10 – 3.83] | 0.0387* |
| **TMT B** |  |  |  | 0.01  [-0.01 – 0.04] | 0.36 |
| **Antidepressant medication** |  |  |  | -3.47  [-7.46 – 0.53] | 0.08 |
| ^a^: Covariates: Duration, CGI-S, TMT-B, and antidepressant | | | | | |

Group difference**:** the mean difference in the OCI-R scores if pans neg. has the average value.

Group * PANSS negative: the difference in OCI-R, that is added when one point of the clinical factor is added, shown with and without covariates being considered
Abbreviations: CI: Confidence interval; CGI-S: Clinical Global Impression Scale; Coeff: Coefficient; PANSS: Positive and Negative Symptoms Scale; TMT: Trail-Making-Test

Article Title: Negative Symptomatology and Clozapine-Induced Obsessive-Compulsive Symptoms: A Cross-Sectional Analysis

Journal Name: European Archives of Psychiatry and Clinical Neuroscience,

Authors: Phillip Kleymann^[[1]](#footnote-1)^a^[[2]](#footnote-2)^, Carla Morgenroth^1^, Stefan Gutwinski^12^, Felix Bermpohl^1^^[[3]](#footnote-3)^, Daniel Schulze^[[4]](#footnote-4)^, Elias Wagner^[[5]](#footnote-5)^^[[6]](#footnote-6)^, Alkomiet Hasan^4^^[[7]](#footnote-7)^, Cynthia Okhuijsen-Pfeifer^[[8]](#footnote-8)^, Jurjen J. Luykx^8^, Marte Z. van der Horst^7^, Tatiana Oviedo-Salcedo^[[9]](#footnote-9)^, Stefanie Schreiter^1^

1. a Corresponding author, phillip.kleymann@charite.de [↑](#footnote-ref-1)
2. Department of Psychiatry and Neurosciences, Charité - Universitätsmedizin Berlin, Corporate Member of Freie Universität Berlin and Humboldt-Universität zu Berlin, Berlin, Germany. [↑](#footnote-ref-2)
3. Department of Psychiatry and Psychotherapy, St. Hedwig-Krankenhaus, Charité-Universitätsmedizin, Berlin, Germany. [↑](#footnote-ref-3)
4. Charité - Universitätsmedizin Berlin, Corporate Member of Freie Universität Berlin and Humboldt-Universität zu Berlin, Institute of Biometry and Clinical Epidemiology, Berlin, Germany [↑](#footnote-ref-4)
5. Department of Psychiatry, Psychotherapy and Psychosomatics, University of Augsburg, Augsburg, Germany. [↑](#footnote-ref-5)
6. Evidence-based psychiatry and psychotherapy, Faculty of Medicine, University of Augsburg, Augsburg, Germany. [↑](#footnote-ref-6)
7. DZPG (German Center for Mental Health), partner site München/Augsburg, Augsburg, Germany. [↑](#footnote-ref-7)
8. Department of Psychiatry, University Medical Center Utrecht, Utrecht University, Utrecht, The Netherlands. [↑](#footnote-ref-8)
9. Department of Psychiatry and Psychotherapy, University Hospital-LMU Munich, Munich, Germany. [↑](#footnote-ref-9)
